# Safety and Efficacy of Dalbavancin in Real Life: Retrospective Analysis of a Large Monocentric Case Series of Patients Treated for Skin/Soft Tissue and Other Difficult-to-Treat Infections

**DOI:** 10.3390/antibiotics13111063

**Published:** 2024-11-08

**Authors:** Giustino Parruti, Ennio Polilli, Simona Coladonato, Giorgia Rapacchiale, Francesca Trave, Elena Mazzotta, Martina Bondanese, Francesco Di Masi, Davide Recinelli, Serena Corridoni, Alberto Costantini, Stefano Ianniruberto, Pierluigi Cacciatore, Fabrizio Carinci

**Affiliations:** 1 Infectious Disease Unit, Santo Spirito General Hospital, 65124 Pescara, Italy; coladonatosimona@gmail.com (S.C.); giorgia.rapacchiale@asl.pe.it (G.R.); trave.francesca@gmail.com (F.T.); elena.mazzotta@asl.pe.it (E.M.); martina.bondanese@ausl.pe.it (M.B.); francesco.dimasi@asl.pe.it (F.D.M.); davide.recinelli@asl.pe.it (D.R.); stefano.ianniruberto@asl.pe.it (S.I.); 2 Clinical Pathology, Santo Spirito General Hospital, 65124 Pescara, Italy; en.polilli@gmail.com; 3 Pharmacy Unit, Santo Spirito General Hospital, 65124 Pescara, Italy; serena.corridoni@asl.pe.it (S.C.); alberto.costantini@asl.pe.it (A.C.); 4 Internal Medicine Day Hospital, Santo Spirito General Hospital, 65124 Pescara, Italy; pierluigi.cacciatore@asl.pe.it; 5 Department of Statistical Sciences, University of Bologna, 40126 Bologna, Italy; fabrizio.carinci@unibo.it

**Keywords:** dalbavancin, infections, tolerability, Gram-positive infections, antibiotic therapy

## Abstract

**Background:** Dalbavancin is a long-acting lipoglycopeptide, approved for treatment of skin and skin structure infections. Its PK/PD profile and safety allow for short hospital stays even in the case of difficult-to-treat infections requiring long courses of therapy, e.g., osteomyelitis, cardiovascular, and prosthetic infections. **Objectives:** We aimed to evaluate the safety and efficacy of dalbavancin in real life settings for both in-label and off-label indications. **Methods:** retrospective evaluation of all consecutive patients treated with dalbavancin at our site between May 2017 and September 2021. Results: A total of 100 patients treated with dalbavancin and followed up for 6 months after treatment (58% male; median age 63.5 years, median Charlson Comorbidity Index CCI = 2.7, 28% inpatients) were included with the following indications: acute bacterial skin and skin structure infections (22%), bone and prosthetic infections (57%), and cardiovascular infections (19%). Infections were caused by MSSA (30%), MRSA (5%), MR-CoNS (20%), and *Streptococcus* spp. (8%). In 32 cases, no isolate was obtained. The average number of infusions was 5 (s.d. = 3). Neither ensuing alteration of renal function nor neutropenia or thrombocytopenia were observed during treatment and follow-up. Two self-limiting skin rashes occurred. The overall clinical success rate was 84%—91% for registered and 82% for unregistered indications. The prescription of higher loading doses was the only predictor independently associated with better outcomes in multivariate models (OR: 5.2, 95%CI: 1.5–17.9, *p* < 0.01). **Conclusions:** Dalbavancin proved to be effective for skin and skin structure infections, as well as for difficult-to-treat infections in highly comorbid patients. Regarding tolerability, our results support the use of dalbavancin for long-lasting treatments of deep-seated infections.

## 1. Introduction

Dalbavancin is a new lipoglycopeptide antibiotic approved by both the FDA and the EMA for the treatment of acute bacterial skin and skin structure infections (ABSSSI). It is characterized by a long half-life, a favorable PK/PD profile, with high tissue and biofilm penetration, and an excellent in vitro and in vivo activity against most Gram-positive pathogens, including methicillin-resistant *Staphylococcus aureus* (MRSA) [1,2,3].

Registrative trials, prevalently enrolling young patients with ABSSSI, ushered in a promising safety profile, with little renal and hematological toxicity [4]. Owing to its favorable PK/PD profile, dalbavancin requires only two infusions to treat ABSSSIs [5], an attractive option for outpatient parenteral antimicrobial therapy (OPAT). Its use may be potentially helpful for long-term treatments that would otherwise require inpatient admissions, avoiding unnecessary in-hospital stays [6,7]. As a consequence of such favorable features, clinicians worldwide showed interest in its use in a wider range of infections caused by Gram-positive bacteria shortly after dalbavancin licensure [8], including osteomyelitis, periprosthetic infections, and endocarditis, which are more complex than ABSSSI due to metafocal bacterial seeding in bones, valves, and periprosthetic tissues and complicated by biofilm production [9]. These infections are often resistant to other available antimicrobial treatments, with a high rate of partial response or relapse [10,11]. Low vascularity, lowered pH, and redox potential restrict the access of antibiotics and immune system components to damaged tissues. In infected indwelling prostheses, the organisms are usually integrated into protective biofilms, making them less susceptible to antibiotics and the host’s immune response [10,11,12]. Intravenous antibiotics such as β-lactams, vancomycin, teicoplanin, and daptomycin are usually administered to patients with deep-seated infections, requiring hospitalization or daily hospital accesses, central line placement, and therapeutic drug monitoring [13]. The PK/PD profile of dalbavancin appears favorable to overcome all such limitations [14,15,16,17,18,19,20,21,22,23]. In recent years, the scientific literature paved the way for a larger off-label use of dalbavancin for unregistered indications, due to the consistently high rates of clinical and microbiological success reported in patients with endocarditis, prosthetic joint infections, and osteomyelitis [16,17,18,19,20]. However, the scarcity of information on the tolerability and efficacy of dalbavancin in real-life settings still hampers its use by clinicians through off-label prescription [21,22].

In this report, we present the clinical results of a 4-year experience with dalbavancin in a consecutive case series of difficult-to-treat infections.

## 2. Results

The general characteristics of the selected cohort are shown in Table 1.

A total of 100 patients were treated in the study period with a full course of dalbavancin prescribed by clinicians. Their age ranged between 20–90 years (median: 63; IQR: 52–76), with 59% being males. The median Charlson Comorbidity Index was equal to 2.7 (IQR: 1–4). The most frequent underlying comorbidities were cardiovascular disease (51%), obesity (24%), and diabetes mellitus (15%). Seventy-five patients were treated as outpatients.

### 2.1. Infections and Microorganisms

The main microorganisms isolated in enrolled patients are shown in Table 2.

Overall, prosthetic joint infections (32%), ABSSSI (22%), spondylodiscitis (13%) and endocarditis (11%) were the most common conditions treated with dalbavancin.

All documented infections were monomicrobial (except for one). The most common isolated pathogens were methicillin sensitive *S. aureus*, methicillin resistant coagulase-negative Staphylococci and *Streptococcus* spp. Other Gram-positive microorganisms that were isolated and treated included *Aerococcus urinae* (1) and *Gemella sanguinis* (1). No microbiological isolate was obtained for 32 patients, among which 25 failed a previous antibiotics treatment.

### 2.2. Source Control, Previous and Combination Treatment

Source Control, previous and combination treatment are shown in Table 3.

Source control surgery of infection was performed in 26 cases (26%) in advance or during treatment with dalbavancin. Proper source control surgery was proposed for 20 patients with prosthetic joint infections, in accordance with current international guidelines for late prosthetic infections [23,24]. Among them, only 12 provided their consent for surgical removal of the prostheses.

Patients who refused surgery were informed of the reduced chances of treatment success and consequently treated with dalbavancin, in association with the best tolerable drug among doxycycline, cotrimoxazole, rifampin or levofloxacin.

A total of 52 patients without source control were prescribed a second or third antibiotic in association with dalbavancin. A total of 86 patients had a history of previous treatments. The trial dose of teicoplanin was omitted for choice of the prescribed clinician in 5 patients.

### 2.3. Treatment Outcomes

**Figure 1:**
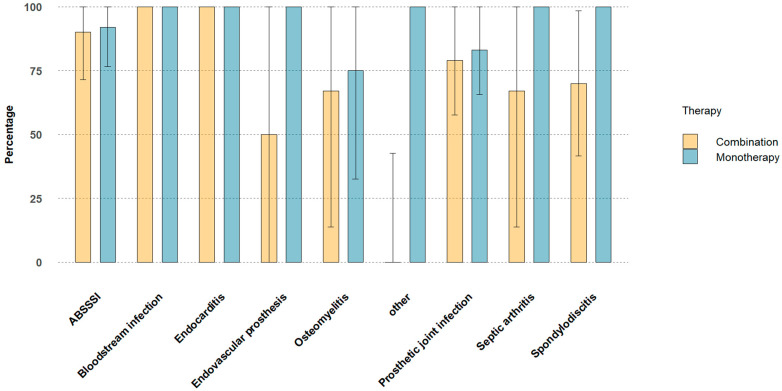
Clinical success rates among different types of infections.

Dalbavancin was well tolerated: we registered two skin rashes considered as mild adverse events, one of which occurring in a patient without previous administration of teicoplanin, which was resolved by short courses of oral steroids and antihistamines. None of the patients with normal or mildly reduced renal function before treatment suffered any GFR reduction possibly related to treatment at any time during follow-up. Similarly, white blood cell counts, including neutrophil counts, were unmodified during treatment and follow-up for all patients. None of the included patients died neither during treatment nor during the additional six months of follow up. Overall clinical success rate was 84%. Clinical success rates according to different types of infection are shown in Figure 1.

**Figure 2:**
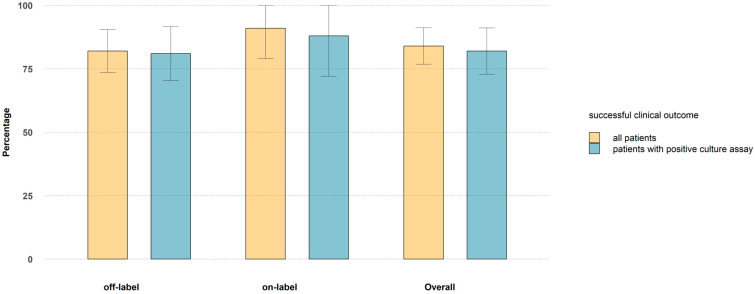
Clinical success rates for registered and unregistered indications, calculated on the whole sample (yellow bars) and on patients with culture proved infections (blue bars).

The rate of success in ABSSSI was 91%, as among the 22 patients receiving dalbavancin for the approved indication, a total of 20 presented a complete resolution of lesions, without relapses by the end of follow up. Such favorable clinical outcome was higher than the 82% scored for all unregistered indications (see Figure 2).

Persistent clinical success at the end of follow-up was recorded for 26 out of the 32 patients with prosthetic joint infections (81%), in 4 of 5 patients for septic arthritis (80%), in all 4 patients with bloodstream infections (100%) and in all 11 patients with endocarditis (100%), in 3 of the 4 patients with endovascular prosthesis infection (75%), in 5 of the 7 patients with osteomyelitis (71%), in 10 of the 13 patients with spondylodiscitis (77%). In the subset of patients with culture proven infections, success rates were overlapping (Figure 2).

Furthermore, one patient treated for an infected breast implant healed, whereas one patient treated for mediastinitis progressed early in his course, unfortunately in line with expectations [25]. No tendency to different response rates appeared between patients treated for the first time or retreated for failure or relapse after a previous treatment.

The results of univariate and multivariate analyses exploring the association between potential predictors and failure rates are shown in Table 4 and Table 5, respectively. Odds of failure in the subset of culture proven infections are shown in parallel to those of the whole sample in both tables.

Previous exposure of 86 patients to antibiotics did not predict any association with failure in the univariate analyses (OR: 1.17, 95%CI: 0.23–5.79, *p* = 0.84). Among the 30 patients with MSSA infections receiving dalbavancin, 24 (80%) had clinical success, as did 4 (80%) of the 5 patients with MRSA infection. Among the 20 patients with methicillin-resistant coagulase-negative staphylococcal infections (MR-CoNS), 18 patients (90%) experienced disease resolution. Out of the 10 patients with *Streptococcus* spp., 8 (80%) similarly experienced clinical success.

As patients with ABSSSI received a loading dose of 1500 mg of dalbavancin, we wondered whether differences in the loading dose might have played a major role in causing different failure rates among patients treated for on-label and off-label indications. To adjust for the potential bias induced by the clinical heterogeneity of enrolled patients, we considered all the main potential confounders of the relationship between dose, indication, and treatment outcomes. From the 16 failing patients, 6 of whom had no microbiological isolate ahead of dalbavancin treatment, control microbiological cultures were obtained for 5 patients, all confirming the same sensitivity profile as for the previous isolate.

Results presented in Table 5 show that none of the other characteristics considered (age, sex, CCI, number of infusions, in-label or off-label indications) are independently associated with the final outcome.

Logistic regression showed that a full-blown loading dose of 1500 mg may decrease the risk of therapy failure by over five-fold (OR: 5.18, 95%CI: 1.50–17.93, *p* < 0.01). The main adjustment covariates, age (OR: 1.03, 95%CI: 0.97–1.09, *p* = 0.35) and male gender (OR: 1.17, 95%CI: 0.35–3.87, *p* = 0.81), were not significantly associated. Among potential predictors, neither in-label versus off-label indications (OR: 0.38; 95%CI: 0.06–2.43, *p* < 0.27), CCI index > 1 (OR: 0.47, 95%CI: 0.06–3.85, *p* < 0.9), nor the number of infusions (OR: 0.88; 95%CI: 0.64–1.21, *p* < 0.93) were significantly associated.

## 3. Discussion

Our investigation aimed to retrospectively investigate the outcomes of dalbavancin use in the treatment of ABSSSI and other difficult-to-treat infections during the first 4 years at our site. We administered dalbavancin to many more patients with other potentially susceptible Gram-positive infections than to those with ABSSSI [2].

Our results confirm high success rates for patients with ABSSSI, in line with the results of both registrative and other recent studies [5,6,7,15]. Moreover, our observational case series provided additional evidence that dalbavancin may be safely used in real-life settings in patients with other difficult-to-treat infections, in line with its well characterized ability to block the synthesis of the Gram-positive bacterial cell wall with a peculiar mechanism of action [1], penetrating into bone, cartilages, and difficult tissues, and reaching biofilm inhibiting concentrations [15,16,17,18,19,25].

From a health care perspective, a large proportion of patients with serious infections and high comorbidity could be treated as outpatients, without experiencing any serious adverse events [26]. Furthermore, in spite of the fear of possible treatment-emergent resistance in failing patients, none of the control isolates obtained after failure displayed a worsened resistance profile [27,28]. Longer treatments were administrated to obtain eradication in patients with difficult-to-treat infections, as in most cases of endocarditis, osteomyelitis, spondylodiscitis, and prosthetic infections [17,20].

Although we did not control C trough concentrations of dalbavancin before further administrations, we considered on the basis of the literature data available at the start of the study [29,30] that further 500 mg doses could be administered under prudent and tight monitoring of possible ensuing toxicity. Our safety results support this practice, even for at-risk patients. Consequently, we are planning to monitor dalbavancin concentrations during prolonged treatments to possibly increase efficacy and reduce costs.

The present results, based on a significant fraction of comorbid and elderly patients, may also suggest that a prolonged use of dalbavancin may be feasible and effective for difficult-to-treat infections with limited alternative treatment options [21,31].

In addition, higher loading doses of dalbavancin were independently associated with better outcomes in difficult-to-treat infections. Evidence for this was already provided by registrative studies and by further recent research on dalbavancin, suggesting that the effect of this drug may well be both dose and time dependent [5,32], especially in patients with long lasting infections and likely biofilm formation at infection site(s) [33]. As for therapeutic drug monitoring, it will be interesting to assess whether repeated higher doses may yield higher success rates when using dalbavancin alone in rescue treatments of infections e.g., chronic osteomyelitis or spondylodiscitis with perivertebral infections [34,35].

Late periprosthetic infections of the elderly showed an unexpected rate of long-lasting clearance. Removal or two-step substitutions of hip or knee prostheses in elderly patients are often risky, with high relapse rates and frequent complications [36,37]. A relevant proportion of patients with periprosthetic infections in our series, in spite of refusing source control surgery, obtained clinical and biochemical infection clearance, suggesting that a conservative treatment with dalbavancin might be offered to frail elderly patients with chronic periprosthetic infections under such circumstance [38,39,40].

There are several limitations in our study; first of all, it is retrospective and monocentric in nature, without any comparator arm or historical control group for both ABSSSI and other difficult-to-treat infections to ensure a better comparison of safety and other treatment outcomes. Indeed, none of the patients died during treatment or follow-up, suggesting a possible selection bias, in spite of reported comorbidities. However, the enrollment of our case series was consecutive, and no patient treated at our site with dalbavancin since 2017 was excluded, thereby providing an estimate of efficacy and safety of dalbavancin in a setting of patients who had mostly failed previous antibiotic treatment(s).

Second, the population of patients in the case series was heterogeneous, as we enrolled patients with different infections, often adding a second or third antibiotic to dalbavancin treatment in pre-treated patients.

Third, we used a trial dose of teicoplanin in advance of dalbavancin administration. This may have reduced the incidence of allergic or idiosyncratic reactions in our cohort. However, our trial dose of teicoplanin did not significantly limit the estimation of renal and hematological tolerability of dalbavancin, which was frequently administered beyond labelled schedules.

## 4. Methods

### 4.1. Study Design

This monocentric retrospective cohort was observed at the Infectious Diseases Unit of “Santo Spirito” Hospital in Pescara, in the Abruzzo Region of Italy. On 3 May 2017, the local health administrative board introduced dalbavancin into the local therapeutic armamentarium by authorizing both the in-label and off-label prescription of dalbavancin at the inpatient ward and the outpatient Infectious Diseases Unit. The administrative board mandated a yearly review of treatment results to monitor dalbavancin expenditure and performance vs. the potential reduction in the length of hospital stays and the annexed costs of hospitalization.

The prescriptions were authorized with the aim of facilitating the early discharge of inpatients or the direct outpatient treatment of serious infections due to Gram-positive pathogens.

Physicians from the Infectious Diseases Unit reviewed their treatment proposal with the head of the Unit prior to starting off-label treatments. Tailoring treatments for longer durations than those indicated in on-label schedules was similarly discussed in advance and based on the clinical and laboratory history and type of infection. Patients considered for treatment with dalbavancin were those consulted at the Unit after failing to respond to previous conventional treatments for ABSSSI, osteomyelitis, endocarditis, and prosthetic joint and endovascular infections. Previous antibiotic treatment(s) were defined as any prescription of antibiotic(s) for the same indication, regardless of the length of treatment and means of administration until the reported interruption. Patients with severe ABSSSI or other indications were also considered for first-line dalbavancin, especially during the COVID-19 period. Patients treated for off-label indications were requested to provide their written informed consent to be treated with dalbavancin, while those treated with a prolonged schedule were asked to sign an additional form for the same specific purpose.

The observational cohort included all consecutive patients treated with dalbavancin between May 2017 and September 2021 for both on-label and off-label indications.

Medical records were retrospectively collected by three Infectious Diseases physicians using electronic formats. In addition to age, sex, and Charlson Comorbidity Index and surgical treatment(s), we registered microbiological data, e.g., site of infection, type of microorganism, susceptibility pattern of bacterial isolates, surgical source control (debridement and implant retention, or implant removal, where performed), and laboratory tests (total blood cell count, neutrophils and platelets absolute counts, hemoglobin levels, urea and creatinine values). Patients were followed up either at outpatient visits (89 patients) or by phone calls (11 patients). All patients were evaluated by either means for persistent or relapsing fever, persistent or relapsing alterations of C-reactive protein, and persistent or relapsing joint pain on charging (when appropriate), as well as for hospital readmissions. Follow-up data at six months after the end of treatment were available for all of them.

Data on other antibiotics administered before and/or concomitant to dalbavancin, the dosage of dalbavancin, the frequency of administration, and length of treatment were similarly collected.

As for antimicrobial therapy prescription, physicians from the Infectious Diseases Unit reviewed their treatment proposal with the head of the Unit prior to starting off-label treatments; tailoring treatments to be longer than those indicated in on-label schedules was also discussed in advance and based on clinical and laboratory history and the type of infection. In 32 patients without any blood or site microbiological evidence of Gram-positive infection, dalbavancin was prescribed, in the absence of any other alternative etiology, for the following minor evidences: clinical diagnosis of ABSSSI in at-risk patients (6 patients, 18.8%); presence of anti-staphylococcal serum antibodies, which correlate with recent and ongoing infection (15 patients, 46.9%) [41]; and microbiological evidence of either streptococcal or staphylococcal infection ahead of previous failing treatments (11, 34.3%).

### 4.2. Primary and Secondary Outcomes

Primary outcomes included measures that were considered relevant for the safety and tolerability of dalbavancin, in terms of severe or mild adverse events attributable to the treatment, particularly with regard to renal function (levels of creatinine; onset of renal injury according to KIDGO criteria), hematologic abnormalities (neutropenia, thrombocytopenia, anemia), gastrointestinal symptoms (diarrhea, nausea, vomiting), and skin rash. Adverse events were classified according to the definition and grading of the World Health Organization.

The secondary outcome was to evaluate clinical efficacy and failure rates in on-label vs. off-label treatment. Clinical cure was defined as resolution of signs and symptoms of infection and normalization of C = reactive protein. Adequate control of the infection source was defined as surgical or radiological procedure(s) aimed to remove the source of infection (ex. abscess drainage or debridement). Treatment failure was defined as a lack of clinical improvement or evidence of persistent infection during treatment. Relapse was defined as a resurgence of clinical and laboratory signs of infection during follow-up.

### 4.3. Dalbavancin Administration

Dalbavancin was administered as a loading dose of 1500 mg, followed by an additional 500 mg a week later in most cases of ABSSSI, in accordance with the in-label schedule. A trial of 1 dose of intravenous (IV) teicoplanin was prescribed to nearly all patients in advance of treatment with dalbavancin. This choice was based on the structural similarities between teicoplanin and dalbavancin to detect possible idiosyncratic or allergic reactions in advance of administration of dalbavancin, which has a much longer half-life than teicoplanin. The dosage, duration, and frequency of dalbavancin administration were tailored for patients with off-label prescriptions. The loading dose of dalbavancin was 1500 mg for osteomyelitis, followed by a second dose of 1500 mg a week later in most cases. Further weekly 500 mg doses were administrated in cases with slow clinical and/or biochemical responses, as well as in cases lacking control of the infection source.

Patients with endocarditis were treated with a loading dose of 1000 mg and additional 500 mg doses, which were prescribed until microbiological, biochemical, and radiological evidence of eradication of the infection was obtained. Some of these prolonged schedules were chosen in accordance with refs. [17,29]. For prosthetic infections in elderly patients with unremovable infected joint prostheses, the loading dose was prudentially decreased to 500 mg, followed by 500 mg weekly administrations for as long as necessary until clinical and biochemical evidence of infection cure was obtained. No further dose adjustments, however, were made for patients with mild renal impairment (CLCr > 30 mL/min). Dalbavancin was prescribed in combination with other antibiotics, such as rifampin, cotrimoxazole, doxycycline or fluoroquinolones, both for inpatient without microbial isolates, to broaden the spectrum of the antibiotic regimen, and for inpatients with previous treatment failure(s), to increase bacterial killing into the biofilm. Other antibiotic(s) in combination were interrupted within 10 days after the last administration of dalbavancin. None of the included patients were left on long-term suppressive treatment.

### 4.4. Statistical Analysis

Descriptive analysis included calculation of the mean and standard deviation for continuous variables, and absolute and relative frequencies for categorical variables. Logistic regression was used to compute univariate and multivariate odds ratios (OR) to explore the level of association between categorical variables and target outcomes. Results were presented in terms of odds ratios with 95% confidence intervals. Multivariate models were adjusted for all relevant retrieved demographic and clinical characteristics.

Predictive factors were included in a fully automated four-step backward elimination process. Age and gender were forced in all models, with all other variables sequentially excluded in three consecutive rounds using a *p* value ≥ 0.20, ≥0.10, and ≥0.05 [42]. Significant values were highlighted by *p*-values < 0.05 [43]. All statistical analyses were carried out using the R language [44].

## 5. Conclusions

Our retrospective evaluation of a large real-life case series adds to the notion that dalbavancin may be considered an effective option for many difficult-to-treat, deep-seated infections other than ABSSSI, with valuable outcomes even in comorbid patients. The tolerability of dalbavancin allowed long-lasting treatments without significant impact on renal function or neutrophil counts. Most patients were treated as outpatients, reducing total hospital stays in a healthcare system under increasing financial pressure.

Larger prospective studies are needed to further define the efficacy of dalbavancin in infections other than ABSSSI, and to explore the potential of therapeutic drug monitoring in these settings.

## Figures and Tables

**Table 1:** General characteristics of study population at baseline (N = 100), and source control and association therapy.

Continuous Variables	Median (IQR)
Age	63.0 (52–76)
Charlson Comorbidity Index	2.7 (1–4)
N. of dalbavancin infusions *	5.2 (4–7)
Categorical Variables	N
Male	59
Obesity (BMI > 30)	24
IDU	2
Diabetes mellitus	15
Cardiovascular disease	51
Pulmonary disease	10
CNS disease	9
HIV	1
Other immunodeficiencies	6
Autoimmune diseases	3
Chronic gastrointestinal disease	7
Solid tumors	3
Hematological malignancies	6
Mild chronic renal insufficiency	8
Hepatic cirrhosis	1
Surgery in the previous three months	14
Charlson Comorbidity Index > 1	71
Trial of teicoplanin dosing	95
Adequate control of the infection source	26
Previous antibiotic treatment	86
High dose	16
Association therapy	53
Cotrimoxazole **	26 (49%)
Rifampicin **	17 (32%)
Doxycyclin **	18 (34%)
Levofloxacin **	1 (2%)

* N = 100; percentages are equal to absolute numbers, ** N (% referred to those with association therapy).

**Table 2:** Distribution of patients by type of infection and causative micro-organisms isolated (N = 100).

Infection Type	Overall	MSSA *	MRSA *	CoNS *	*Streptococcus* spp.	Other **	Negative Culture
ABSSSI	22	10	2	3	1		6
Prosthetic joint infection	32	6	2	6	2		16
Septic arthritis	5	2	0	1	0		2
Bloodstream infections	4	2	0	2	0		0
Endocarditis	11	0	0	5	4	2 **	0
Endovascular prosthesis	4	0	0	0	1		3
Osteomyelitis	7	3	1	0	1		2
Spondylodiscitis	13	5	0	3	1		4
Other	2	2	0	0	0		0

* MSSA: methicillin-susceptible, *Staphylococcus aureus*; MRSA: Methicillin-resistant *Staphylococcus aureus*; CoNS, Coagulase-negative *staphylococci*. ** *Gemella sanguinis*, *Aerococcus* spp.

**Table 3:** Antibiotics associated with dalbavancin in combination therapy.

Infection Type	Source Control (n)	TMP-SMX	RMP	DOX	LVX and AZM	DOX and RMP	DOX and TMP-SMX	DOX and RMP	DOX and MET
ABSSSI		1	4	3		2			
BSI			2				1		
Endovascular prosthesis		2							
Endocarditis			4		1		1		
Osteomyelitis	Debridement (2)			3					
Septic arthritis	Drainage (5)	1		1				1	
PJI	Two-stage revision arthroplasty (12)	7	2	2			1	1	1
Spondylodiscitis	Percutaneous drainage of paravertebral effusion (4) Surgical debridement (3)	5		5					

TMP-SMX: Trimethoprim/sulfamethoxazole; RMP: Rifampicin; DOX: Doxycyclin; LVX: Levofloxaciclin; AZM: Azithromycin; MET: metronidazole; ABSSSI: Acute Bacterial Skin and Skin Structure Infections; BSI: Bloodstream Infections; PJI: Prosthetic Joint Infection.

**Table 4:** Univariate analysis for prediction of dalbavancin for treatment failure.

Variable	OR (95%CI)	*p* > *χ* ^2^	OR (95%CI) *	*p* * > *χ* ^2^
Age	1.02 (0.98–1.05)	0.33	1.04 (1.00–1.08)	0.06
Male	0.87 (0.30–2.57)	0.81	0.78 (0.22–2.77)	0.70
Autoimmune disease	2.73 (0.23–32.08)	0.45	NA	0.06
BMI ≥ 30	0.40 (0.08–1.91)	0.21	0.25 (0.03–2.09)	0.14
Charlson’s comorbidity score > 1	1.27 (0.37–4.33)	0.70	1.94 (0.47–7.97)	0.34
Monotherapy vs. combination	2.20 (0.70–6.88)	0.16	1.39 (0.37–5.18)	0.62
Diabetes	0.33 (0.04–2.73)	0.24	NA	0.03
Hematological malignancy	2.86 (0.48–17.11)	0.28	2.45 (0.20–29.50)	0.50
Heart Disease	0.95 (0.33–2.78)	0.93	1.24 (0.36–4.32)	0.74
Immunosuppression	1.05 (0.11–9.67)	0.96	1.61 (0.15–16.92)	0.70
Neurologic disorder	0.63 (0.07–5.44)	0.66	0.93 (0.10–8.74)	0.95
On-label/Off-label	0.46 (0.10–2.18)	0.29	0.60 (0.12–3.08)	0.52
500 mg vs. 1500 mg loading dose	5.00 (1.61–15.57)	<0.01	5.22 (1.37–19.90)	0.02
Respiratory tract Disease	1.36 (0.26–7.07)	0.72	2.04 (0.35–12.03)	0.45
Source control	0.94 (0.27–3.22)	0.92	1.15 (0.30–4.33)	0.84
Previous antibiotic therapy	1.17 (0.23–5.79)	0.85	1.57 (0.17–14.11)	0.67
Number of infusions	1.05 (0.81–1.36)	0.70	0.95 (0.70–1.28)	0.73
Endovascular prosthesis	1.80 (0.18–18.49)	0.64	NA	
Osteomyelitis	2.26 (0.40–12.80)	0.38	1.18 (0.12–11.62)	0.89
Septic arthritis	1.33 (0.14–12.77)	0.81	2.45 (0.20–29.50)	0.50
Prosthetic joint infection	1.34 (0.44–4.07)	0.61	1.65 (0.43–6.39)	0.47
Spondylodiscitis	1.71 (0.41–7.05)	0.47	1.40 (0.25–7.76)	0.71
ABSSSI	0.46 (0.10–2.18)	0.29	0.60 (0.12–3.08)	0.52
Other	5.53 (0.33–93.38)	0.26	5.00 (0.29–86.13)	0.29
Culture proven infection	1.50 (0.44–5.08)	0.51	-	-
MSSA	1.50 (0.49–4.59)	0.48	1.33 (0.38–4.65)	0.65
*Streptococcus* spp.	1.36 (0.26–7.07)	0.72	1.20 (0.22–6.52)	0.83
CoNS	0.52 (0.11–2.52)	0.39	0.42 (0.08–2.13)	0.26
Other micro-organisms	0.87 (0.10–7.73)	0.89	0.76 (0.08–6.94)	0.80

* After excluding 32 patients with negative microbiological results. NA, not available (not all categories were available to compute OR).

**Table 5:** Results of multivariate logistic regression (outcome: treatment failure).

Variable	OR (95%CI)	*p* > *χ* ^2^	OR (95%CI) *	*p* * > *χ* ^2^
Age	1.03 (0.97–1.09)	0.35	1.05 (0.98–1.13)	0.12
Males	1.17 (0.35–3.87)	0.81	1.10 (0.25–4.78)	0.70
On-label/Off-label	0.38 (0.06–2.43)	0.27	0.34 (0.04–2.97)	0.50
CCI > 1	0.47 (0.06–3.85)	0.90	0.43 (0.04–5.09)	0.43
Number of infusions	0.88 (0.64–1.21)	0.93	0.72 (0.46–1.12)	0.42
500 mg vs. 1500 mg leading dose	5.18 (1.50–17.93)	<0.01	5.64 (1.15–27.75)	0.01

CCI: Charlson Comorbidity Index. * After excluding 32 patients with negative microbiological results.

## Data Availability

The data that support the findings of this study are available on request from the corresponding author.
